# Editorial: Recent Advances and Technologies in Algal Lipid Biology

**DOI:** 10.3389/fpls.2016.01444

**Published:** 2016-09-27

**Authors:** Jeffrey Leblond, Jeremy Dahmen

**Affiliations:** ^1^Department of Biology, Middle Tennessee State UniversityMurfreesboro, TN, USA; ^2^Matrix GeneticsSeattle, WA, USA

**Keywords:** algae, lipids, sterol, fatty acid, biofuel

Within this research topic entitled “Recent Advances and Technologies in Algal Lipid Biology” is a collection of five papers which demonstrate the breadth and depth of current algal lipid research. Before highlighting the findings of these five papers, it is worth stepping back to define the terms “algae/algal” and “lipid” as they relate to these studies, especially because these terms are commonly used in the popular scientific press associated with the push to develop algae and their lipids as a source of renewable fuel.

A lipid is defined as any biological compound which is soluble in a non-polar organic solvent, such as chloroform, and generally insoluble in water. According to this definition, lipids represent arguably the most diverse macromolecule class because they can include such molecules as fatty acid-containing lipids (e.g., glycolipids, betaine lipids, phospholipids, and triglycerides), sterols (e.g., cholesterol), hydrocarbons (e.g., *n*-alkanes and alkenes), pigments (e.g., chlorophylls, bacteriochlorophylls, and carotenoids), and toxins (e.g., brevetoxins produced by the dinoflagellate *Karenia brevis*). As such, the papers within this research topic focus on fatty-acid containing lipids and sterols.

Algae are generally considered to be aquatic, non-plant, photosynthetic organisms which, along with plants, are primary producers that fix CO_2_ and produce the O_2_ crucial to the survival of consumer organisms. However, this definition is too narrow because many eukaryotic algae are non-photosynthetic, hypothesized as having lost their plastid en route to a heterotrophic life cycle. Furthermore, although most algae are unicellular, several such as kelps and other seaweeds are multicellular. Although most live in true aqueous environments, some such as members of the green algae and the cyanobacterial genus *Gloeocapsa* live, respectively, in environments subject to intense heat and drying such as the crust of desert soil and the roofs of houses (Lewin, 2006; Karsten and Holzinger, 2014). The algae examined by the papers within this research topic are all aquatic, photosynthetic, and some are possible targets for biofuel production.

Within the photosynthetic algae covered by this set of papers, certain lipids have specific roles within cells. For example, depending on the type of fatty acid-containing lipid, it can serve as either the basis for a cellular membrane (i.e., glycolipids, betaine lipids, and phospholipids) or as an energy storage molecule (i.e., triglycerides). More specifically, glycolipids such as monogalactosyldiacylglycerol (MGDG), digalactosyldiacylglycerol (DGDG), and sulfoquinovosyldiacylglycerol (SQDG) comprise the chloroplast membrane matrix within which photosynthetic pigments such as chlorophylls and carotenoids reside, whereas betaine lipids and phospholipids are thought to form other cellular membranes. Many of these lipids contain potentially economically valuable, long-chain, polyunsaturated fatty acids (Guschina and Harwood, 2006). Sterols are considered to reinforce betaine lipid- and phospholipid-based cellular membranes in most eukaryotes (Duforc, 2008). Within this research topic, Sato and Awai provide a brief overview of the biosynthesis of MGDG, DGDG, and SQDG, and discuss how the biosynthetic pathways have evolved from the earliest photosynthetic prokaryotic life forms with particular emphasis on how glycolipid biosynthesis in cyanobacteria differs from that in the chloroplasts of algae and plants.

Because of a cell's need to maintain membrane integrity throughout a range of environmental conditions, lipid composition is often modulated according to variables such as temperature and irradiance. Accordingly, two of the papers within this research topic focus on these variables. In the work by Anesi et al. it was shown how the effect of growth temperature on freshwater dinoflagellates leads to discrete differences in glycolipid fatty acid composition, and may explain how and why certain dinoflagellates inhabit particular environmental niches. The work of Wacker et al. demonstrated for a variety of eukaryotic algae that irradiance stimulates changes in fatty acid composition. However, this response is carried out differently across different species, leading to the conclusion that each alga responds uniquely to this particular environmental condition. These types of studies are important because they not only give insight as to how algae are able to survive environmental conditions which change from a daily to a seasonal scale, but also how growth conditions may be modified to induce algae to produce high levels of biofuel lipids.

With regard to using algae to produce biofuel, the paper by Gu et al. is an example of current technology whereby a lipid biosynthesis gene from one alga may be introduced into another alga (that is easier to grow on a commercial scale) as a means of producing more lipid. This highlights how a given algal species will probably need to be genetically augmented to make it a more efficient producer of lipid. Specifically, Gu et al. utilized a type III ketoacyl-ACP synthase from a halophilic diatom, *Chaetoceros* sp. isolated from Great Salt Lake, to replace an analogous enzyme in the cyanobacterium *Synechococcus* sp.; this led to improved production of medium-chain fatty acids (e.g., C_14:0_). Medium- chain fatty acids are of particular interest as biofuels because they may be more suitable in colder temperatures than longer chain fatty acids.

Lastly, within the realm of algal lipids, there is a long history of using “unique” lipids as biomarkers for particular genera or classes of algae. The work of Taipale et al. is a continuation in a long history of studies (see seminal paper by Volkman et al., 1998) which demonstrate how fatty acids, and particularly sterols, may be used to resolve algal species within a community assemblage.

In summary, algae and lipids demonstrate a diversity of cell types and macromolecules, respectively. The set of papers in this research topic has given insight into how current experimental approaches can be used to both assess lipid functionality and improve their production in algae.

## Author contributions

All authors listed, have made substantial, direct and intellectual contribution to the work, and approved it for publication.

### Conflict of interest statement

The authors declare that the research was conducted in the absence of any commercial or financial relationships that could be construed as a potential conflict of interest.
